# 3′-*O*-β-Glucosyl-4′,5′-didehydro-5′-deoxyadenosine Is a Natural Product of the Nucleocidin Producers *Streptomyces virens* and *Streptomyces calvus*

**DOI:** 10.1021/acs.jnatprod.3c00521

**Published:** 2023-09-25

**Authors:** Xuan Feng, Qingzhi Zhang, David J. Clarke, Hai Deng, David O’Hagan

**Affiliations:** † School of Chemistry, University of St Andrews, North Haugh, St Andrews, KY16 9ST, U.K.; ‡ EaStChem School of Chemistry, University of Edinburgh, Joseph Black Building, David Brewster Road, Edinburgh, EH9 3FJ, U.K.; § Department of Chemistry, University of Aberdeen, Aberdeen, AB24 3UE, U.K.

## Abstract

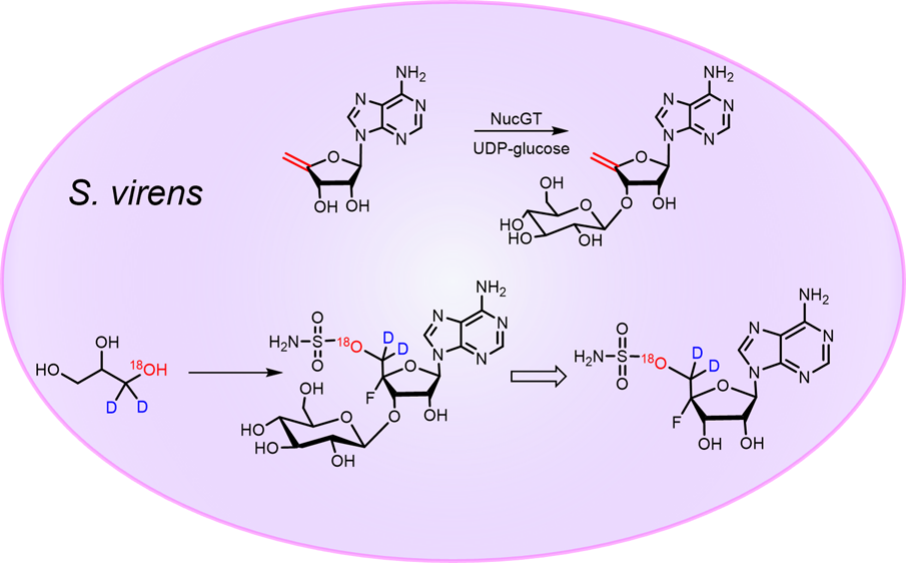

3′-*O*-β-Glucosyl-4′,5′-didehydro-5′-deoxyadenosine **13** is identified as a natural product of *Streptomyces calvus* and *Streptomyces virens*. It is also generated *in vitro* by direct β-glucosylation of 4′,5′-didehydro-5′-deoxyadenosine **12** with the enzyme NucGT. The intact incorporation of oxygen-18 and deuterium isotopes from (±)[1-^18^O,1-^2^H_2_]-glycerol **14** into C-5′ of nucleocidin **1** and its related metabolites precludes 3′-*O*-β-glucosyl-4′,5′-didehydro-5′-deoxyadenosine **13** as a biosynthetic precursor to nucleocidin **1**.

Nucleocidin **1** is a modified nucleoside antibiotic isolated originally from *Streptomyces calvus*.^1^ It is of structural interest as a natural product because it contains a fluorine atom and a sulfamyl moiety, two functional groups that are exceedingly rare in nature.^2^ This has to be contrasted with the widespread use of selective fluorination and to a lesser extent sulfamylation, in medicinal chemistry and drug discovery programs.^3^ Genome mining has allowed several additional Streptomyces strains to be identified which also have the ability to produce nucelocidin **1** and various related metabolites in culture.^4^ These organisms include *Streptomyces virens*, which is a good producer of nucelocidin **1**. Only a handful of fluorine-containing metabolites are known, the most notable of which is fluoroacetate **2**, a toxin found in a wide range of plants and bacteria.^5^ A bacterial fluorination enzyme (fluorinase) that converts *S*-adenosyl-l-methionine (SAM) to 5′-fluorodeoxyadenosine (5′-FDA **3**) is known to be involved in bacterial fluoroacetate **2** biosynthesis; however, that enzyme is not involved in nucleocidin biosynthesis.^6^ There is no such fluorinase gene encoded in any of the genomes of the nucleocidin producers, and the site of the fluorine atom at the 4′-carbon of the ribose is inconsistent with the chemistry of that enzyme.

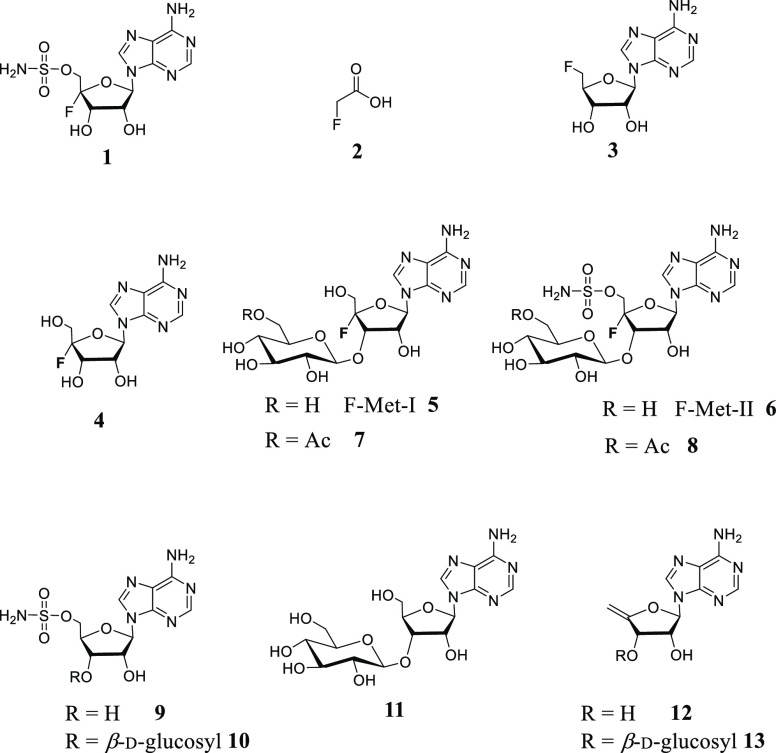



In an effort to shed light on nucelocidin **1** biosynthesis, we and others have identified additional fluorinated metabolites associated with nucleocidin **1**.^7^ These include 4′-fluoroadenosine **4** and F-Met I **5** and F-Met II **6**, the latter two of which are β-glucosylated at the 3′-O hydroxy group.^7a^ Most recently, the acetylated glucose derivatives **7** and **8** were also reported.^7b^ We and others^7,8^ have carried out extensive gene knockout experiments probing the putative nucleocidin biosynthetic gene cluster. We reported^8^ recently that at least 11 genes are required for sulfamylation, and four genes appear crucial to fluorination. Interestingly when sulfamylation ability was disabled in selected KO strains, this did not always adversely affect F-Met-I **4** production, and conversely some gene KOs that disabled fluorination still resulted in 5′-*O*-sulfamylated natural products such as **9** and **10**. Some of these findings are reinforced in the studies of Zechel et al.^7c^ Consistent with the observed decoupling of these biosynthetic motifs, we have found that the corresponding nonfluorinated, but sulfamylated metabolites **9** and **10** are also co-produced with nucleocidin **1** and its analogues in the wild-type producing strains of *S. calvus* and *S. virens* at a level of about 10–15% of the fluorometabolites.^7b^

## Results and Discussion

We now report the isolation of 3′-*O*-β-glucosyl-4′,5′-didehydro-5′-deoxyadenosine **13** from both *S. calvus* and *S. virens*. 3′-*O*-β-Glucosyl-4′,5′-didehydroadenosine **13** was observed initially by mass spectrometry (412.17 Da) from the total ion chromatogram of crude natural product extracts of *S. calvus*. In order to confirm the structure of **13** by isolation, cultures of both *S. calvus* and *S. virens* were grown to maturity (see SI), and nucleocidin **1** and its related metabolites such as **5** and **6** were isolated as previously described^7^ after adsorption onto charcoal and then washing with acetone. HPLC fractionation guided by MS-MS analysis was used to identify 3′-*O*-β-glucosyl-4′,5′-didehydro-5′-deoxyadenosine **13**, and the metabolite was further isolated in low microgram amounts after two rounds of preparative HPLC. High-resolution mass spectrometry (HRMS) gave an accurate mass for **13** ([M + H]^+^ = 412.1451 *m*/*z*, C_16_H_22_N_5_O_8_^+^, Figure S3). Sufficient material (around 0.2 mg) was isolated to be able to record ^1^H NMR. In addition, a reference sample of **13** was prepared from synthetic **12** following a previously reported synthesis.^9^

With synthetic **12** in hand, we explored its enzymatic 3′-O-glucosylation using a previously identified^7a^ glucosyl transferase, NucGT, which is associated with the nucleocidin **1** biosynthetic gene cluster (BGC). NucGT from *S. calvus* and *S. virens* is already shown to have the ability to β-glucosylate the 3′-OH group of nucleocidin **1**, adenosine, and defluoronucleocidin **9** to generate **6**, **10**, and **11**, respectively. Compound **12** was assayed as a potential substrate for NucGT, and it generated a product that proved to be identical to **13** by HPLC retention time and the ^1^H NMR of the isolated product was essentially identical to semisynthetic **13**. The data are listed in Table 1. The alignment of the two ^1^H NMR spectra is shown in the SI (Figure S4).

**Table 1:**
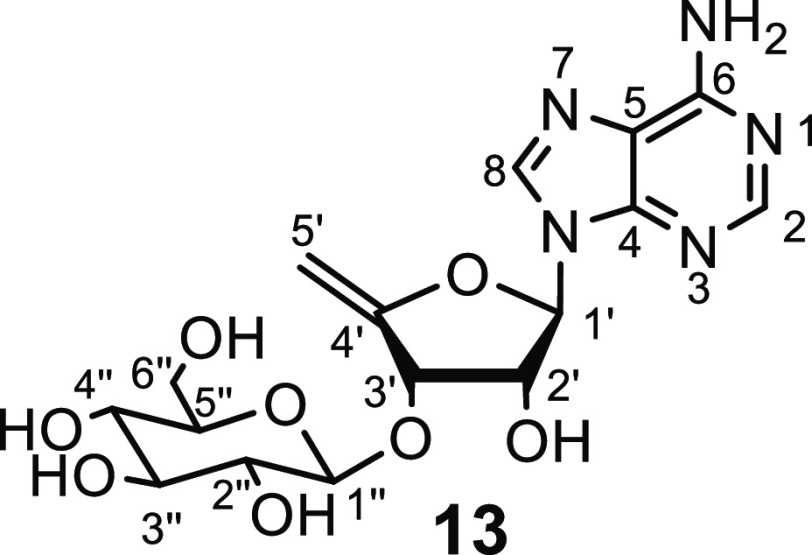
^1^H NMR (700 MHz, δ in ppm, *J* in Hz, in *d*_6_-Acetone) Comparison of Natural and Semisynthetic 3′-*O*-β-Glucosyl-4′,5′-didehydro-5′-deoxyadenosine **13**

	**13** (natural)	**13** (semisynthetic)
position	δ_H_	δ_H_
2	8.22 (s)	8.22 (s)
8	8.26 (s)	8.27 (s)
1′	6.30 (d, 5.2)	6.32 (d, 5.4)
2′	5.12 (q, 5.4)	5.13 (t, 5.1)
3′	5.20 (d, 4.9)	5.17 (d, 4.9)
5′a	4.51 (dd, 3.5, 2.2)	4.52 (t, 1.4)
5′b	4.45 (d, 1.7)	4.45 (t, 1.2),
1″	4.69 (d, 7.8)	4.67 (d, 7.8)
2″–5″	3.39–3.50 (m)	3.35–3.48 (m)
6″ H^a^	3.69 (m)	3.69 (m)
6″ H^b^	3.85 (d, 6.2)	3.87 (m)

**Figure 1:**
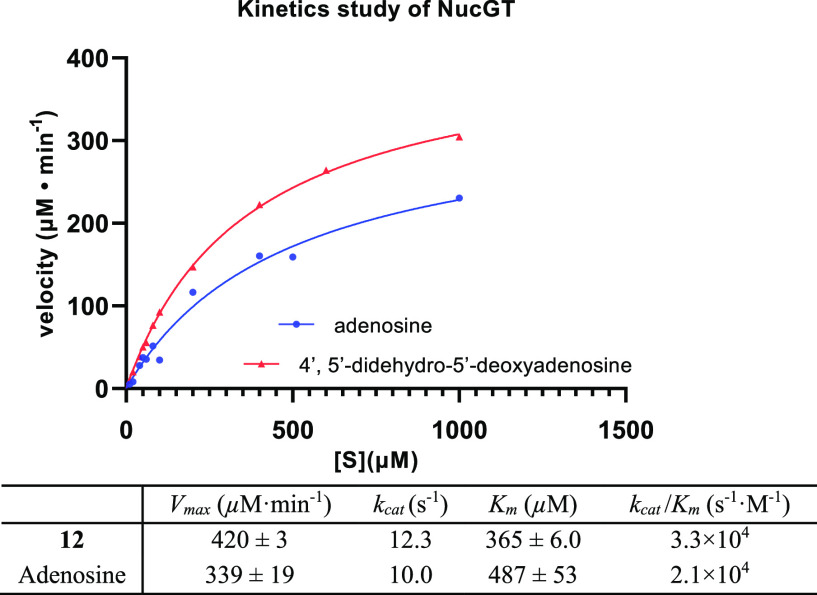
Kinetic data of NucGT with 4′,5′-didehydro-5′-deoxyadenosine **12** and adenosine.

A study of the reaction kinetics for NucGT with **12** and UDP-glucose to generate **13** evaluated key kinetic parameters (*K*_m_ and *V*_max_), and the data presented in Figure 1 unexpectedly indicated that **12** is a measurably more efficient substrate overall than adenosine.

The presence of **13** in the culture medium and the ability of NucGT to convert **12** to **13** presented the possibility that either **13** or **12** may be a biosynthetic precursor to nucleocidin **1**. For example, epoxidation of the 4′,5′-exocyclic double bond and then epoxide ring opening with fluoride ion at C-4′ presented a plausible strategy for fluorination at C-4′ of the ribose with concomitant formation of the necessary C–O bond at C-5′. It follows that if this C–O bond is introduced late in the biosynthesis from molecular oxygen, then the oxygen atom would not derive from the original ribose, but instead would arise from an oxidizing enzyme supplied by molecular oxygen.^10^ Thus, we decided to carry out an incubation experiment with (±)[1-^18^O,1-^2^H_2_]-glycerol **14**. Glycerol incorporates into the pentose phosphate pathway and is known to contribute an intact C–O bond to ribose at C-5′–O.^11^ Thus, the successful incorporation of both deuterium and oxygen-18 isotopes from glycerol would disqualify **13** and **12** as biosynthetic intermediates to nucleocidin **1**, as retention of an intact C-5′–O bond from glycerol would disqualify an origin by oxidation of the exomethylene double bond.

**Figure 2:**
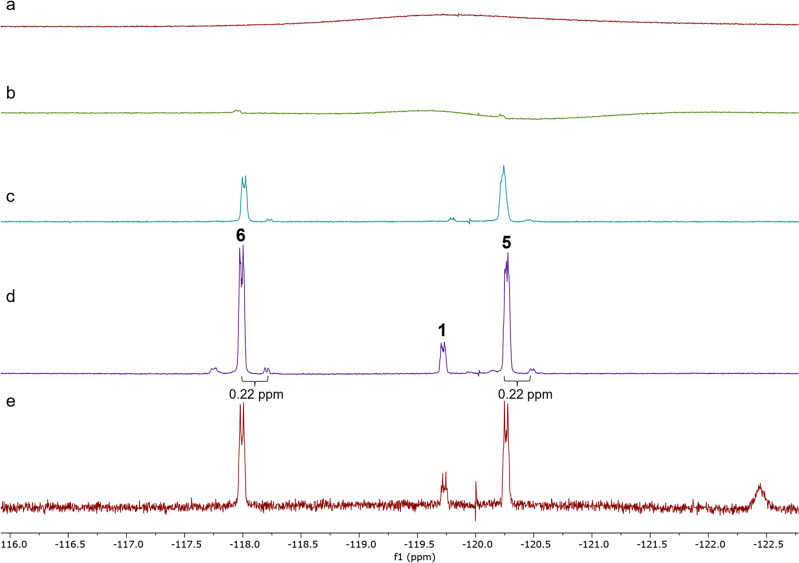
^19^F{^1^H} NMR of *S. virens* extracts with (±)[1-^18^O,1-^2^H_2_]-glycerol **14** pulse supplementation after (a) 3 days, (b) 4 days, (c) 6 days, or (d) 8 days of incubation and (e) *S. virens* 6-day culture with normal glycerol supplementation.

Isotopically labeled (±)[1-^18^O,1-^2^H_2_]-glycerol **14** was prepared by adaption of a previous route describing the syntheses of [1-^13^C,^18^O]- and [1-^13^C,^2^H_2_]-glycerols, but in this case using both oxygen-18 water (97 atom % ^18^O) and sodium borodeuteride (97 atom % ^2^H_2_) in the synthesis.^12^ The resultant (±)[1-^18^O,1-^2^H_2_]-glycerol **14** was estimated by mass spectrometry to be labeled with oxygen-18 at ∼82 atom % and deuterium at 97 atom %. This glycerol was pulse supplied into cultures of *S. virens* (final conc. 8.8 mM), and the production of fluorometabolites was determined by ^19^F{^1^H} NMR. The cultures were harvested after 8 days, and the metabolites were extracted using standardized protocols. The resultant ^19^F{^1^H} NMR spectra for the glycerol **14** supplementation experiments and a control using unlabeled glycerol are illustrated in Figure 2.

**Scheme 1:**
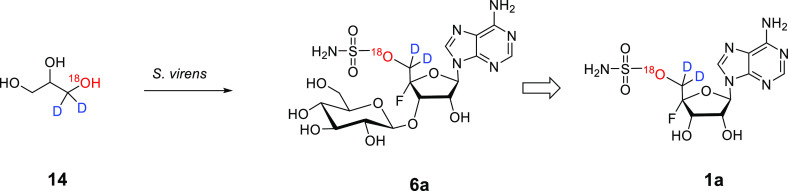
Intact Incorporation of Isotopes from (±)[1-^18^O,1-^2^H_2_]-Glycerol **14**

It is notable that there are clear heavy isotope (deuterium)-induced fluorine signals ∼0.22 ppm upfield of F-Met I **5** and F-Met II **6** at approximately 1–2% of the unlabeled signal. This is entirely consistent with the incorporation of two deuterium atoms into C-5′ as illustrated in Scheme 1 and previously established^11^ in glycerol supplementation experiments, and it is a clear indication of intact incorporation of the deuteriums from (±)[1-^18^O,1-^2^H_2_]-glycerol **14** into C-5′ of the fluorometabolites. It was important now to determine if the oxygen-18 atom was also incorporated, although this could not be determined directly by ^19^F{^1^H} NMR as the chemical shifts induced by ^18^O over ^16^O are just too small to be recorded over three bonds.

The intact incorporation of ^18^O along with both deuterium atoms of **14** was, however, confirmed by LC-HRMS and FT-ICR MS analyses. Metabolite extracts from (±)[1-^18^O,1-^2^H_2_]-glycerol **14**-supplied *S. virens* were semipurified on HPLC. The fractions containing fluorometabolites **5** and **6** were analyzed by LCMS and LC-HRMS, and isotope fine structure analysis was conducted on FT-ICR MS particularly to explore the intensity of any [M + 4 + H]^+^ ions associated with the fluorometabolites. The [M + 4 + H]^+^ abundance for **6** was 2.6% (**14** supplied) relative to the parent unlabeled molecular ion, where it was 0.0% in the control (unlabeled glycerol added, Figure S17). The value of 2.6% is approximately twice that indicated for deuterium incorporations by ^19^F{^1^H} NMR; however there are two sites for glycerol incorporation into the β-glucosylated metabolites; these are the ribose ring and the β-glucose moiety itself. Therefore, daughter ion fragmentation analysis was conducted in an FT-ICR experiment to deconvolute the incorporation of the isotopes into the ribose and glucose moieties. This analysis indicated that there was a 1.0% [M + 4 + H]^+^ abundance of heavy isotope detected from the daughter ion 369.0842 *m*/*z* of F-Met II **6** by FT-ICR MS analysis. This ion contains the ribose moiety but no longer has the β-glucosyl moiety. The level of enrichment is at a level consistent with the observed ^19^F NMR incorporations, and the difference indicates incorporations of the isotope also into the β-glucose moiety of the parent molecule. The intact incorporations of both deuterium and oxygen-18 from the (±)[1-^18^O,1-^2^H_2_]-glycerol **14** supplementation experiments established that **12** and **13** are not biosynthetic precursors to nucleocidin **1**.

**Figure 3:**
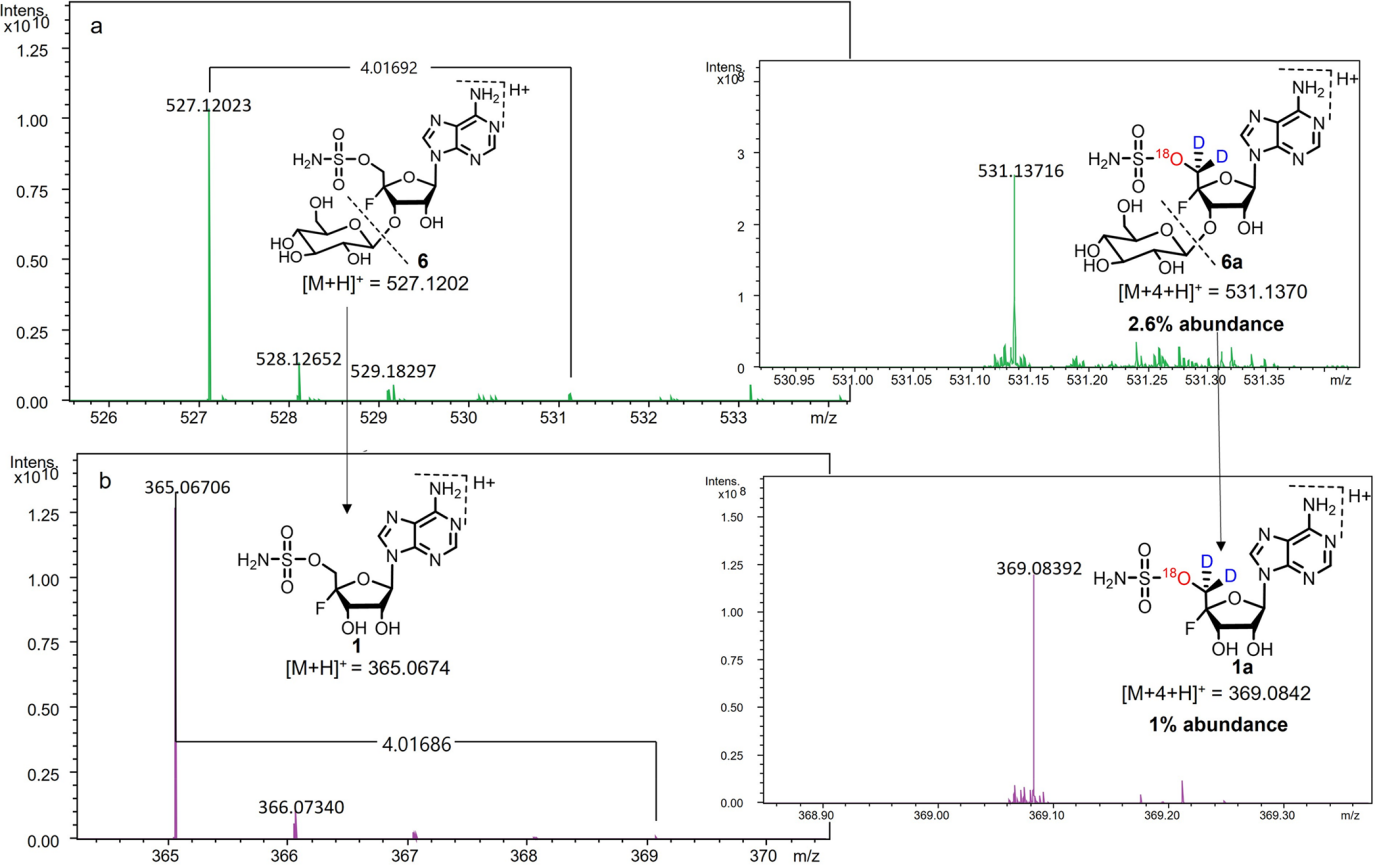
FT-ICR MS-MS analysis of metabolite F-Met II **6** after incubating with (±)[1-^18^O,1-^2^H_2_]-glycerol **14** from *S. virens*. (a) Spectrum of the parent ion showing 2.6% enrichment of [M + 4 + H]^+^ in **6**. (b) Spectrum of the daughter ion (369 amu) of **6** without the glucose attached, indicating 1% enrichment of [M + 4 + H]^+^, suggesting isotope incorporation into the ribose moiety only.

## Conclusions

In summary, we have identified 3′-*O*-β-glucosyl-4′,5′-didehydro-5′-deoxyadenosine **13** as a metabolite of both *S. calvus* and *S. virens*. Supplementation experiments with (±)-[1-^18^O,1-^2^H_2_]-glycerol **14** indicated the intact incorporation of both deuteriums and the oxygen-18 atom into C-5′ of F-Met II **6**, and thus **13** or deglucosylated **12** does not appear to be a biosynthetic precursor of nucleocidin **1** and its related fluorometabolites, as this would be inconsistent with oxygen-18 retention from glycerol. NucGT is able to convert dehydroadenosine **12** to **13***in vitro*, although it is not clear if this is relevant metabolically, as **12** could not be identified in *S. virens* extracts. Such dehydroadenosines have not previously been reported as microbial natural products, although 4′,5′-didehydro-5′-deoxyadenosine **12** has been detected analytically within excreted metabolites as a disease^13^ and dietary^14^ biomarker in mammalian metabolism. In addition, **12** has been identified as a photodegradation product of adenosyl cobalamin *in vitro*,^15^ and also 4′,5′-didehydro-5′-deoxyadenosine **12** is interconverted with adenosine *in vitro* by the action of purified *S*-adenosyl-l-homocysteine hydrolase,^16^ so free dehydroadenosine **12** may get processed that way if **13** is deglucosylated.

## Experimental Section

### General Experimental Procedures

Room temperature refers to 18–25 °C. Air- and moisture-sensitive reactions were carried out under an atmosphere of argon in oven-dried glassware. All evaporations and concentrations were performed under reduced pressure (*in vacuo*) with a Büchi Rotavapor R-200. The freeze-drying was performed under vacuum by a Christ Alpha 1-2 LDplus −55 °C freeze-dryer. All reagents were purchased from commercial suppliers and were used without further purification unless otherwise stated. Anhydrous solvents (DCM, THF, Et_2_O) were obtained from an MBraun MB SPS-800 solvent purification system by passage through two drying columns and dispensed under an argon atmosphere. All microbiological work was carried out in a Gallenkamp laminar flowhood, using standard sterile techniques. Glassware and consumables for biological operations were sterilized by autoclaving, flaming, or wiping with 75% ethanol before using. Sterilized consumables were used as supplied. Media were sterilized by 121 °C, 15 min autoclaving. Cell cultures were incubated in a temperature-controlled incubator (New Brunswick Scientific). Centrifugation of 20 mL to 1 L was processed by a Beckman Avanti centrifuge. A Hettich Mikro 200 benchtop centrifuge was used for microcentrifugation.

### Nuclear Magnetic Resonance (NMR) Spectroscopy

NMR spectra were recorded at 298 K on a Bruker Advance II 400, Advance III HD 500, or Advance III HD 700 instrument. ^1^H and ^13^C NMR spectra were recorded in a deuterated solvent as the lock and the residual solvent as the internal standard. ^19^F NMR spectra were recorded by using CFCl_3_ as an external reference. Chemical shifts are reported in parts per million (ppm), and coupling constants (*J*) are reported in hertz (Hz). The abbreviations for the multiplicity of the proton, carbon, and fluorine signals are as follows: s singlet, d doublet, dd doublet of doublets, ddd doublet of doublet of doublets, t triplet, dt doublet of triplets, q quartet, m multiplet, br s broad singlet.

### LC-MS Analysis

Extracts from culture media were freeze-dried, resuspended in 50% acetonitrile/water to about 1–5 mL, and centrifuged (21300*g*) for 10 min to remove precipitates. These samples were analyzed at the Mass Spectrometry Facility at the University of St Andrews using ThermoFisher Xcalibur Orbitrap instrument in positive ion mode. Due to low abundance of metabolites, some samples were partially purified by HPLC; the majority of the acetonitrile/water elution fractions were collected, and after removal of the solvent, the dry extracts were resuspended in water.

High-resolution electrospray ionization spectra were acquired on a Bruker MaXis II ESI-Q-TOF-MS instrument connected to a Dionex 3000 RS UHPLC instrument fitted with an ACE C4-300 RP column (100 × 2.1 mm, 5 μm, 30 °C). The metabolites were eluted with a linear gradient of 5–100% MeCN containing 0.1% formic acid over 30 min. The mass spectrometer was operated in positive ion mode with a scan range of 200–3000 *m*/*z*. Source conditions: end plate offset at −500 V; capillary at −4500 V; nebulizer gas (N_2_) at 1.8 bar; dry gas (N_2_) at 9.0 L min^–1^; dry temperature at 200 °C. Ion transfer conditions: ion funnel RF at 400 Vpp; multiple RF at 200 Vpp; quadrupole low mass at 200 *m*/*z*; collision energy at 8.0 eV; collision RF at 2000 Vpp; transfer time at 110.0 μs; prepulse storage time at 10.0 μs. MS data were analyzed using Bruker DataAnalysis.

FT-ICR high-resolution MS data were acquired on a 12T SolariX 2XR Fourier transform–ion cyclotron resonance instrument equipped with electrospray (ESI) ionization (Bruker Daltonics). RP-HPLC-purified samples were infused at 2 μL/min, and spectra were acquired between 280 and 4000 *m*/*z* using 4 MWord data collection. Using these conditions, mass resolution of ca. 1,000,000 was achieved, allowing isotope fine structure analysis. For fragmentation experiments, individual species were isolated using the quadrupole, and tandem MS was performed using collision-induced dissociation (CID) by applying a collision energy of 15–25 V.

### Growth of *Streptomyces calvus* and *Streptomyces virens* on Solid Media

*S. calvus* and *S. virens* were grown on solid ISP4 agar plates made with soluble starch (10 g/L), calcium carbonate (2 g/L), ammonium sulfate (2 g/L), sodium chloride (1 g/L), dipotassium phosphate (1 g/L), magnesium sulfate heptahydrate (1 g/L), ferrous sulfate (1 mg/L), manganese chloride (1 mg/L), zinc sulfate (1 mg/L), agar (2%, w/w), and deionized water (to 1 L). The ISP4-agar medium was autoclaved before use. The plates were incubated at 30 °C for 2 to 10 days.

### Seed Culture of *Streptomyces calvus* and *Streptomyces virens*

The seed culture was performed in TSBY liquid medium composed of tryptone soy broth (3%, w/w), sucrose (10.3%, w/w), and yeast extract (0.5%, w/w). The seed cultures of *S. calvus* and *S. virens* were obtained by inoculating 50 μL of spores into 50 mL of TSBY, and the culture was allowed to grow at 28 °C for 2 days (50 mL of medium, in a 250 mL conical flask with shaking at 180 rpm).

### Fermentation Culture

A mass of the mycelium of *S. calvus* or *S. virens* was obtained by inoculating a sterilized, defined medium (100 mL in a 500 mL conical flask) with the seed culture obtained above (inoculate with 2 mL per 100 mL), and the culture was allowed to grow at 28 °C, 180 rpm for 8 days. The defined medium (1 L) was made with tap water, corn steep liquor (12.5 g), mannitol (10 g), sodium chloride (2 g), diammonium phosphate (2 g), monopotassium phosphate (1.5 g), magnesium sulfate heptahydrate (0.25 g), Hoagland’s salt solution (1 mL), and potassium fluoride solution (7.5 mL, 0.5 M).

Hoagland’s salt solution (1 L) contains deionized water, manganese(II) chloride tetrahydrate (0.389 g), phosphorus acid (0.611 g), copper(II) sulfate (0.056 g), ammonium molybdate tetrahydrate (0.056 g), nickel(II) sulfate hexahydrate (0.056 g), zinc sulfate heptahydrate (0.056 g), aluminum sulfate (0.056 g), stannous chloride dihydrate (0.028 g), cobalt(II) nitrate hexahydrate (0.056 g), titanium dioxide (0.056 g), lithium chloride (0.028 g), potassium iodide (0.028 g), and potassium bromide (0.028 g).

### Synthesis of 4′,5′-Didehydro-5′-deoxyadenosine **12**

Compound **12** was prepared following the method reported by P. Perrone.^9^ Iodine (8.86 g, 34.9 mmol) and triphenylphosphine (9.16 g, 34.9 mmol) were added to a solution of adenosine (6.22 g, 23.3 mmol) in pyridine (50 mL) at rt. After 2 h, a saturated solution of Na_2_S_2_O_3_ was added; the solvent was removed under reduced pressure, and the residue was purified by column chromatography using as eluent CHCl_3_/MeOH (9:1). The 5′-deoxy-5′-iodoadenosine (8.5 g, 22.5 mmol, 97%) obtained was dissolved in pyridine (50 mL), ^t^BuOK (11.4 g, 101 mmol) was added, and the mixture was stirred at 80 °C for 1 h. The solvent was removed under reduced pressure, and the residue was purified by column chromatography using as an eluent a mixture of CHCl_3_/MeOH from 9:1 to 7:3. The product was obtained as an off-white solid (4.1 g, 16.5 mmol, 73%): ^1^H NMR (500 MHz, methanol-*d*_4_) δ 8.29 (s, 1H, H-2), 8.23 (s, 1H, H-8), 6.24 (d, *J* = 5.3 Hz, 1H, H-1′), 4.89 (t, *J* = 5.3 Hz, 2H, H-2’), 4.79 (dt, *J* = 5.2, 1.1 Hz, 1H, H-3′), 4.50 (dd, *J* = 2.1, 1.1 Hz, 1H, H-5′a), 4.37 (dd, *J* = 2.0, 0.9 Hz, 1H, H-5′b) (Figures S1 and S2).

### Preparation of 3′-*O*-β-Glucosyl- 4′,5′-didehydro-5′-deoxyadenosine **13** by NucGT

NucGT reaction with 4′,5′-dehydroadenosine **12** was carried out in 50 mM Tris-HCl buffer, pH = 8.0, with 10 mM UDP-glucose, 100 mM MgCl_2_, 2 mM substrate, and 0.567 μM glucosyltransferase enzyme. The reaction was incubated overnight in a heat-block at 37 °C. The reaction mixture was analyzed by HPLC, and the identity of the products was confirmed by LC-MS of isolated fractions.

### Kinetic Study

Enzymatic activity was assayed at 37 °C by monitoring the production using analytical HPLC (Shimadzu SPD-20A detector at 254 nm coupled with a SIL-20A HT autosampler). The glucosyltransferase (0.567 μM) was incubated with various concentrations of substrates, MgCl_2_ (100 mM), and a saturating concentration of UDP-glucose (17.7 mM) in Tris-HCl buffer (50 mM, pH 7.8), in a final volume of 0.25 mL. An aliquot (100 μL) was denaturalized with phenol/chloroform at various time points (1 or 2 min) and then instantly cooled on ice. Precipitated protein was then removed by centrifugation (13 000 rpm, 10 min at 4 °C), and the sample was filtered with a PTFE filter (0.22 μm, Fisherbrand). The eluant was injected into analytical HPLC to determine the level of the products against a standard curve. Each sample was injected three times to obtain the average value. Kinetic parameters were obtained by Michaelis–Menten fitting of the initial velocity against substrate concentrations using Prism 8.0.

### Pulse Supplementation Experiment

Cultures of *S. calvus* or *S. virens* (100 mL) were shaken at 30 °C, labeled glycerol was added after 2 days, and then the same quantity was added every day for the next 6 days. The final concentration of labeled glycerol was 8.8 mM. After 8 days of fermentation, the cells were discarded after centrifugation and the supernatant was extracted by charcoal. The crude extract was analyzed by ^19^F{^1^H} NMR (500 MHz, D_2_O, 4000 scans) to detect fluorometabolites.

### Extraction and Purification of 3′-*O*-β-Glucosyl-4′,5′-didehydro-5′-deoxyadenosine **13**

After 6 to 8 days of incubation, the Streptomyces cells were discarded by centrifugation, and the supernatant was extracted with charcoal/Celite (5 g per 1000 mL). The charcoal/Celite was mixed at a ratio of 1:2. The mixture was stirred in the supernatant for 1 h to absorb natural products. The charcoal/Celite was collected by filtration and then washed by 100 mL of acetone. The acetone was dried *in vacuo*, and the residue was redissolved in deionized water and then fractionated on a Shimadzu LC20A HPLC system with a Phenomenex C18 Luna semipreparative column. MiliQ water was used as mobile phase A, and acetonitrile was used as mobile phase B. Enzymatically prepared **13** was used as a reference for HPLC preparation. A gradient method (0–5 min, 100% A; 15 min, 85% A and 15% B; 25 min, 5% A and 95% B; 35 min, 5% A and 95% B; 36–42 min, 100% A) was applied for the first purification, and an isocratic method (0–15 min,10% mobile phase B and 90% A) was used for the second purification. An analytical HPLC was applied to confirm the retention time (Figure S3). The purified **13** is an off-white solid after freeze-drying (0.2 mg from a 4 L fermentation): ^1^H NMR (700 MHz, acetone-*d*_6_) δ 8.26 (s, 1H, H-8), 8.22 (s, 1H,H-2), 6.30 (d, *J* = 5.2 Hz, 1H, H-1′), 5.20 (d, *J* = 4.9 Hz, 1H, H-3′), 5.12 (q, *J* = 5.4 Hz, 1H, H-2′), 4.69 (d, *J* = 7.8 Hz, 1H, H-1″), 4.51 (dd, *J* = 3.5, 2.2 Hz, 1H, H-5′a), 4.45 (d, *J* = 1.7 Hz, 1H, H-5′b), 3.85 (d, *J* = 6.2 Hz, 1H, H-6″b), 3.69 (m, 1H, H-6″a), 3.50–3.39 (m, 4H, H-2″–5″).

### Purification of Enzymatically Prepared **13**

The purification of **13** was achieved on a Shimadzu LC20A HPLC system with a Phenomenex C18 Luna semipreparative column. The compound was separated with the isocratic method 0–15 min, 10% mobile phase B (acetonitrile) and 90% A (miliQ water). The fraction was then concentrated and freeze-dried for further analysis. The purified **13** is a white solid (1.2 mg): ^1^H NMR (700 MHz, acetone-*d*_6_) δ 8.27 (s, 1H, H-8), 8.22 (s, 1H,H-2), 6.32 (d, *J* = 5.4 Hz, 1H, H-1′), 5.17 (d, *J* = 4.9 Hz, 1H, H-3′), 5.13 (t, *J* = 5.1 Hz, 1H, H-2′), 4.67 (d, *J* = 7.8 Hz, 1H, H-1″), 4.52 (t, *J* = 1.4 Hz, 1H, H-5′a), 4.45 (t, *J* = 1.2 Hz, 1H, H-5′b), 3.90–3.85 (m, 1H, H-6″b), 3.69 (dd, *J* = 11.3, 5.8 Hz, 1H, H-6″a), 3.48–3.35 (m, 4H, H-2″–5″).

### Preparation of (±)-[1-^18^O,1-^2^H_2_]-glycerol **14**.^12^

#### Ethyl [1-^18^O]-3-(benzyloxy)-2-hydroxypropionate

5′,3-Benzyloxy-2-hydroxypropionitrile (1.97 g, 0.43 mmol), prepared according to the literature,^12^ was dissolved in anhydrous ethanol (30 mL). Acetyl chloride (9.9 mL, 139 mmol, 12.5 equiv) was added dropwise over 45 min. The mixture was allowed to warm to rt, and stirring was continued overnight. The volatiles were removed under reduced pressure to yield a white solid. The solid was suspended in dry toluene, and the toluene was removed under reduced pressure. The crude imidate salt was mixed with ^18^O-water (1.0 g, 50 mmol, 4.5 equiv, 97 atom % ^18^O) and dry THF (35 mL) and stirred for 16 h. Water (20 mL) was added, the ester was extracted with EtOAc (3 × 20 mL) and dried (MgSO_4_), and the solvent was evacuated under reduced pressure to give the crude ester as a light brown oil. Column chromatography (silica gel, hexane/ethyl acetate, 4:1 to 1:1) gave the ester a light yellowish oil (1.53 g, 6.77 mmol, 61% over two steps). ^18^O enrichment was 83% as judged by ^13^C NMR and MS. ^1^H NMR (CDCl_3_, 400 MHz) 7.39–7.30 (m, 5H, ArH), 4.65 (d, 1H, *J* = 11.2 Hz, 1/2 CH_2_), 4.56 (d, 1H, *J* = 11.2 Hz, 1/2 CH_2_), 4.33 (t, 1H, *J* = 3.2 Hz), 4.28 (q, *J* = 7.15 Hz), 2.96 (br s, 1H, OH), 1.30 (t, 3H, CH_3_); ^13^C NMR (CDCl_3_, 125 HMz) 172.7 (^13^C=^16^O, 17%), 172.6 (^13^C=^18^O), 137.7, 128.4, 127.8, 127.7, 73.5, 71.4, 70.8, 61.9, 14.2; *m*/*z* (ESI^+^) 249.0977 [M + Na]^**+**^, C_12_H_16_O_3_^18^ONa requires 249.0989 (Figures S6–S10).

#### [1-^18^O,^2^H_2_]-3-Benzyloxy-2-hydroxypropanol

Sodium borodeuteride (NaB^2^H_4_, 98 atom % ^2^H) (1.0 g, 239 mmol, 3.5 equiv) was added in batches to an ice-cooled solution of the preprepared ester (1.53 g, 6.77 mmol) in MeOD (10 mL). The mixture was allowed to warm to room temperature, and stirring was continued over 12 h. The reaction was quenched with saturated aqueous NH_4_Cl (30 mL), and the solution was concentrated on a rotary evaporator to remove MeOH. The aqueous residue was extracted with EtOAc (3 × 30 mL), and the organic layers were combined, washed with brine, and dried (Na_2_SO_4_). After solvent removal, the residue was purified by column chromatography (EtOAc) to give the diol as a colorless oil (1.26 g, 6.77 mmol, quantitative): ^1^H NMR (CDCl_3_, 400 MHz) 7.41–7.30 (m, 5H, ArH), 4.58 (s, 2H, CH_2_), 3.93 (pseudo t, 1H, *J* = 4.0 Hz, CH), 3.64–3.56 (m, 2H, CH_2_), 2.62 (br s, 1H, OH), 2.06 (br s, 1H, OH); ^13^C NMR (CDCl_3_, 125 MHz) 137.7, 128.6, 128.0, 127.9, 73.7, 71.9, 70.5, 63.4 (quintet, CD_2_); *m*/*z* (ESI^+^) 209.1001 [M + Na]^+^, C_10_H_12_D_2_O_2_^18^ONa requires 209.1009 (Figures S11–S14).

#### (±)-[1-^18^O,1-^2^H_2_]-Glycerol

A mixture of the preprepared benzyl ether (1.26 g, 6.77 mmol) and 10% palladium on carbon (677 mg) in methanol (50 mL) was stirred vigorously at room temperature under a hydrogen atmosphere. The reaction was monitored by TLC and was completed after 16 h. The reaction mixture was filtered through Celite followed by washing several times with MeOH. The filtrate was concentrated under reduced pressure to give the product as thick colorless oil (640 mg, 6.67 mmol, 98.5%): ^1^H NMR (CD_3_OD, 400 MHz) 3.66 (t, 1H, *J* = 5.5 Hz), 3.60 (dd, *J* = 5.0, 11.1 Hz), 3.53 (dd, *J* = 6.0, 11.1 Hz); ^13^C NMR (MeOD, 125 MHz) 72.3, 63.0, 62.3 (quintet, *J* 21.2 Hz); *m*/*z* [ESI^+^] 119.0535 [M + Na]^+^, C_3_H_6_D_2_O_2_^18^ONa^+^, requires 119.0539 (Figures S15–S17).

## Supporting Information Available

HRMS and 1D and 2D NMR spectra and experimental materials (PDF)
