# Supplementary material for: 3′-O-β-Glucosyl-4′,5′-didehydro-5′-deoxyadenosine Is a Natural Product of the Nucleocidin Producers Streptomyces virens and Streptomyces calvus
Source: J Nat Prod. 2023 Sep 25;86(10):2326–32. doi: 10.1021/acs.jnatprod.3c00521 (PMC10616807; doi:10.1021/acs.jnatprod.3c00521)
Supplement: Supplementary file 1 — np3c00521_si_001.pdf [file np3c00521_si_001.pdf]

SUPPLEMENTARY INFORMATION

O3'-*β*-Glucosyl- 4', 5'-didehydro-5'-deoxyadenosine is a natural product of the nucleocidin producers *Streptomyces virens* and *Streptomyces calvus*

Xuan Feng,[a] Qingzhi Zhang[a], David J. Clarke,[b] Hai Deng[c] and David O'Hagan[a]*

[a] School of Chemistry, University of St Andrews, North Haugh, St Andrews, KY16 9ST, UK.

[b] EaStChem School of Chemistry, University of Edinburgh, Joseph Black Building, David Brewster Rd, Edinburgh, EH9 3FJ, UK.

[c] Department of Chemistry, University of Aberdeen, Aberdeen, AB24 3UE, UK

INDEX

Pages 2-5 Spectroscopic data of isolated and synthetic O3'-*β*-glucosyl- 4', 5'-didehydro-5'-deoxyadenosine **12.**

Pages 6-12 Preparation of (+/-) [1-^{18}O, 1-$^{2}H_2$]-glycerol **14**.

Page13-14 HRMS analysis of (+/-) [1-^{18}O, 1-$^{2}H_2$]-glycerol **14** supplementation culture extracts

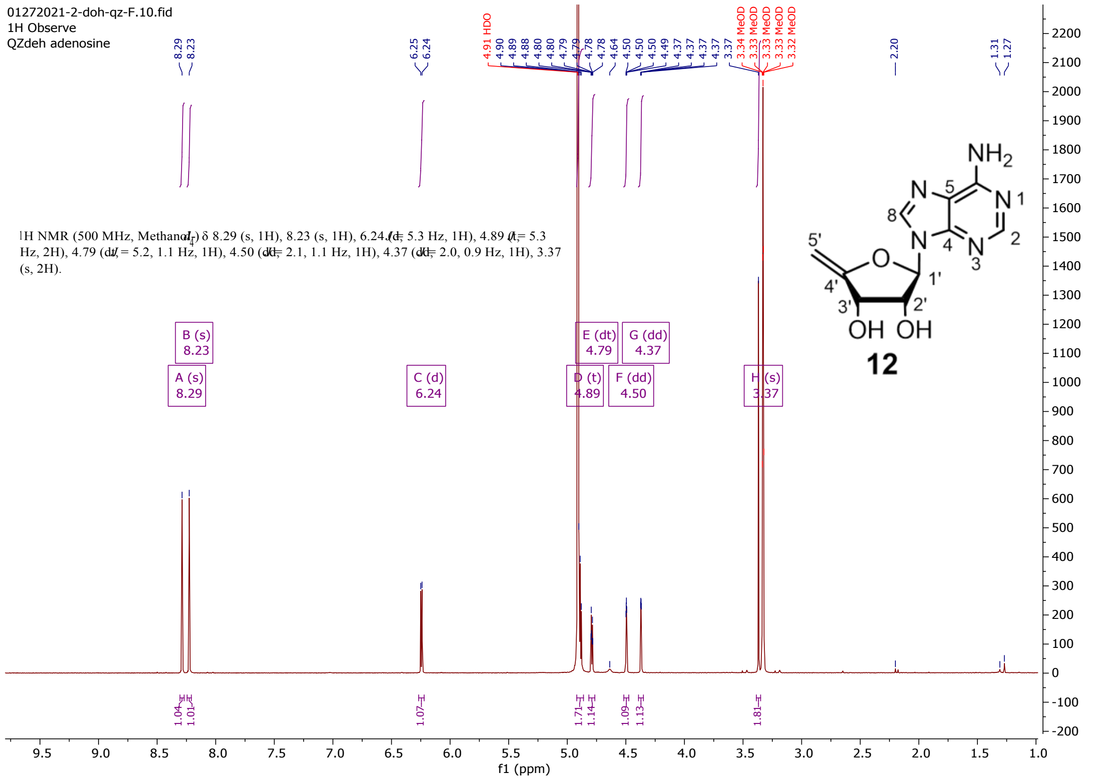

Figure S1. ^{1}H-NMR (500 MHz, Methanol-d_4) of synthetic **12**.

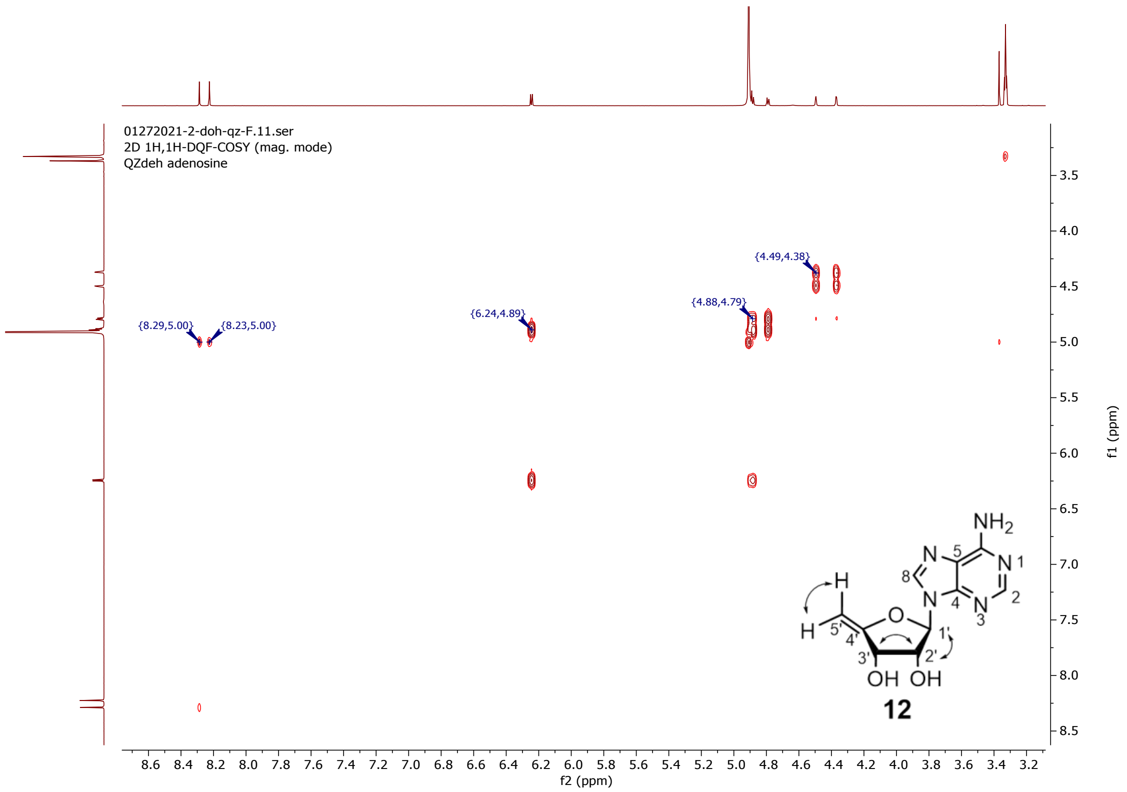

Figure S2. ^{1}H-^{1}H COSY (500 MHz, Methanol-d_4) of synthetic **12**.

Structural elucidation of 3'-O-*β*-glucosyl- 4', 5'-didehydro-5'-deoxyadenosine 13

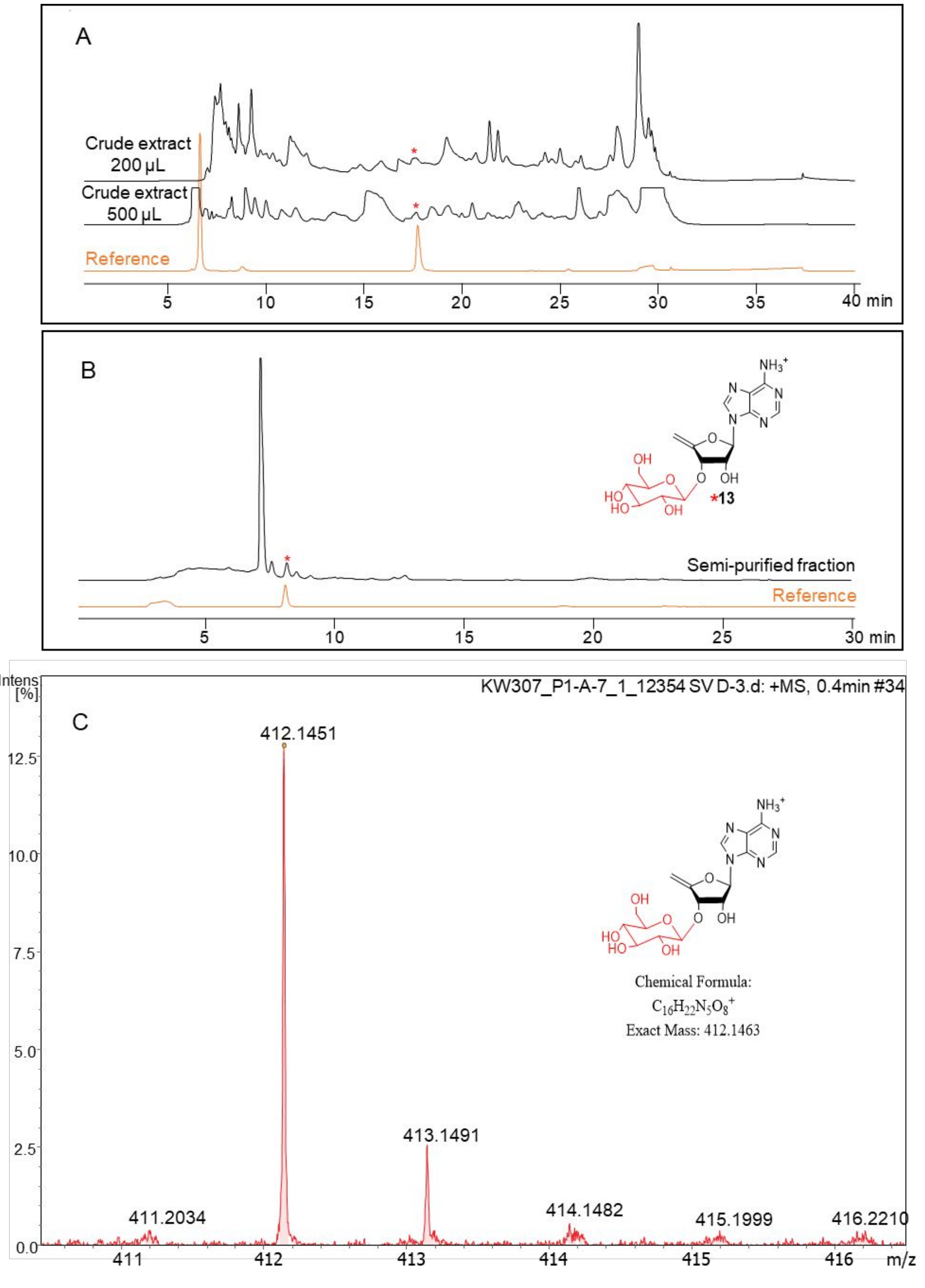

Figure S3. HPLC chromatograms of (A) fractionation of *S. virens*/*S. calvus* crude extract; (B) further purification of the fraction contain **13** and (C) HRMS of natural **13**.

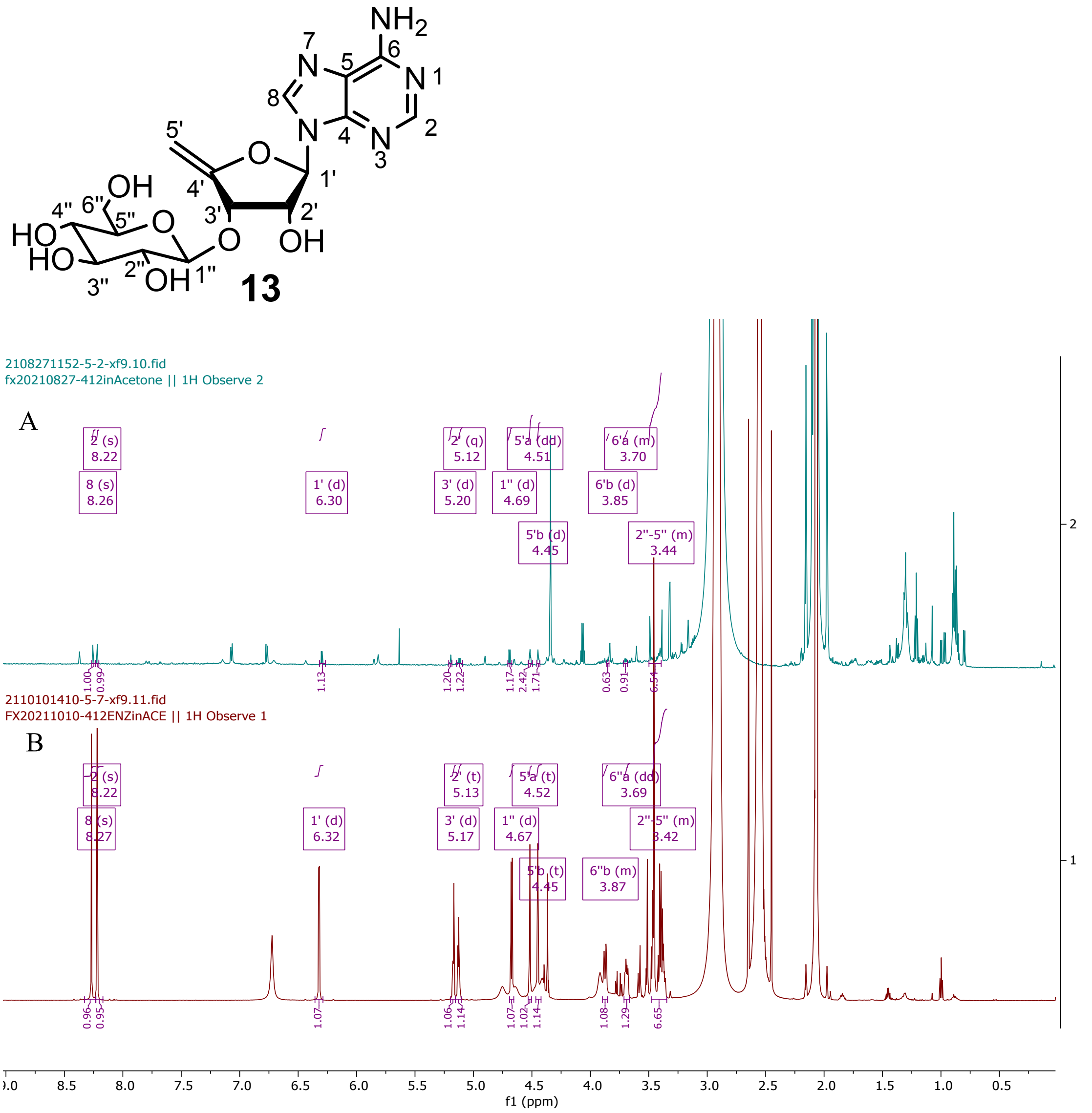

Figure S4. Alignment of ^{1}H-NMR (700 MHz, d_6-acetone) of isolated natural **13** (A) and synthetic-enzymatic **13** (B).

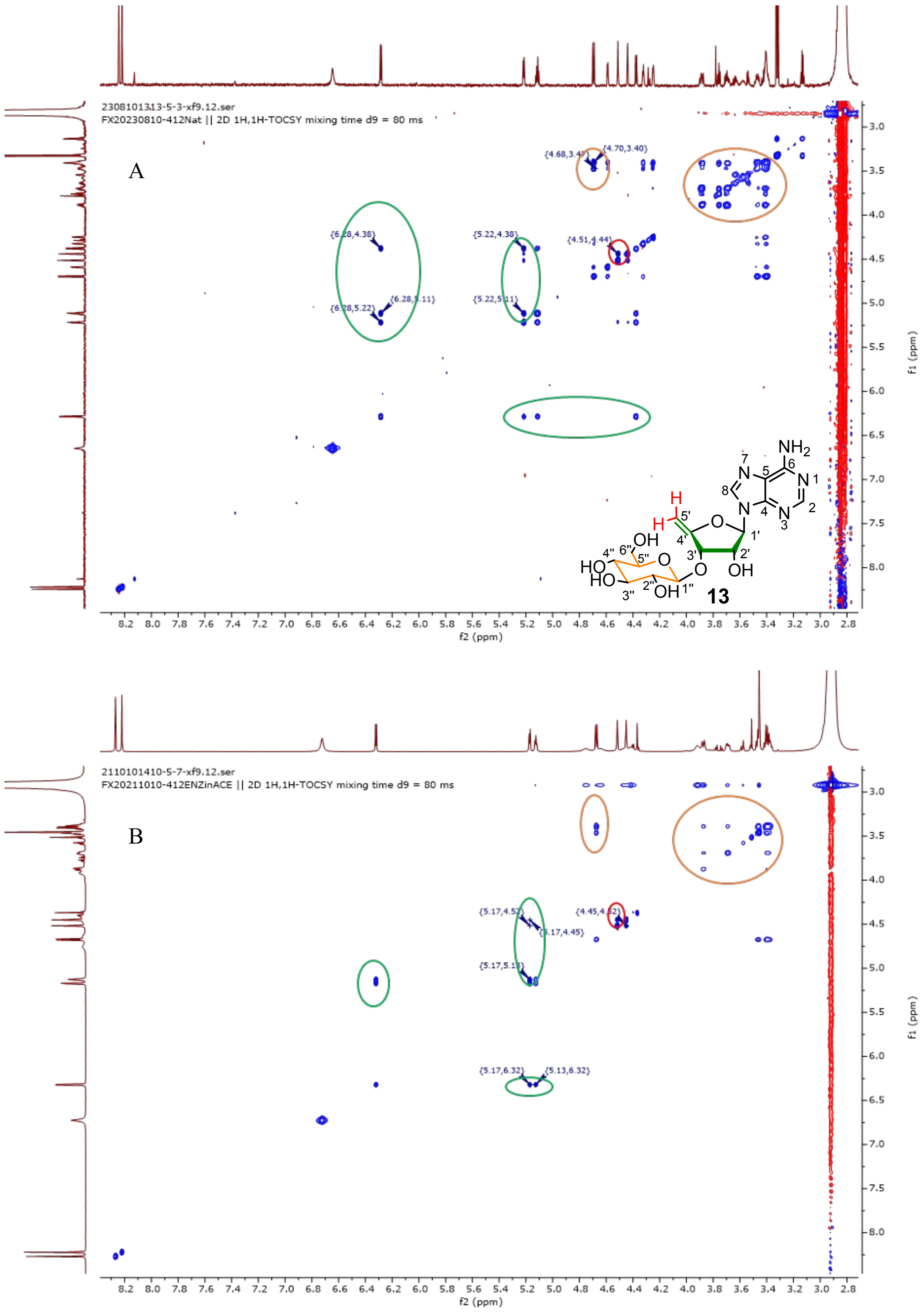

Figure S5. Comparison of ^{1}H-^{1}H total correlation spectroscopy (^{1}H-^{1}H TOCSY) of isolated natural **13** (A) and synthetic-enzymatic **13** (B).

Preparation of (+/-) [1-^{18}O, 1-$^{2}H_2$]-glycerol 14.

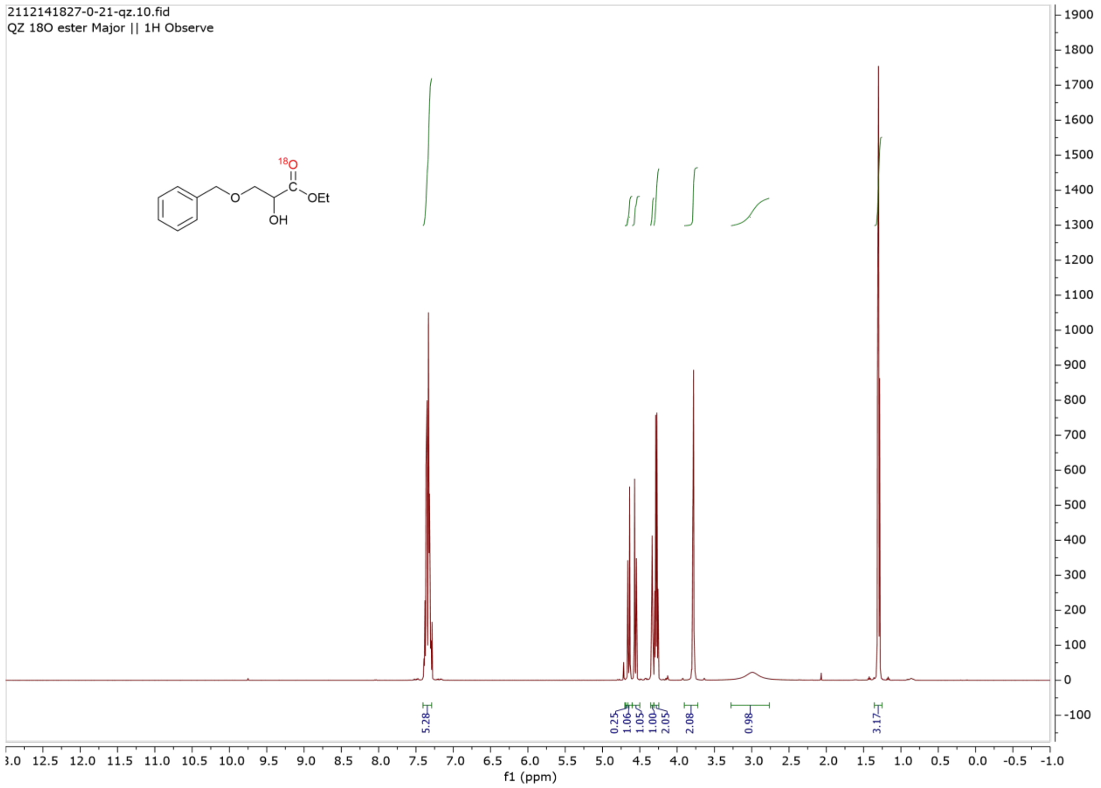

Figure S6. ^{1}H NMR of ethyl [1-^{18}O]-3-(benzyloxy)-2-hydroxypropionate in $CDCl_3$.

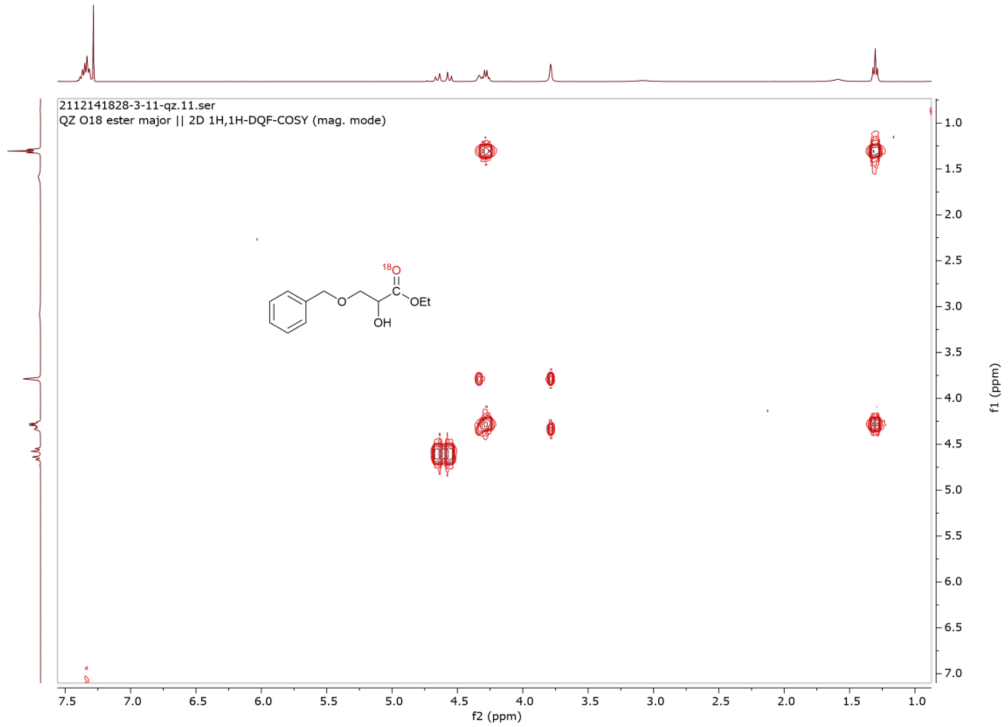

Figure S7. ^{1}H-^{1}H COSY of ethyl [1-^{18}O]-3-(benzyloxy)-2-hydroxypropionate in $CDCl_3$.

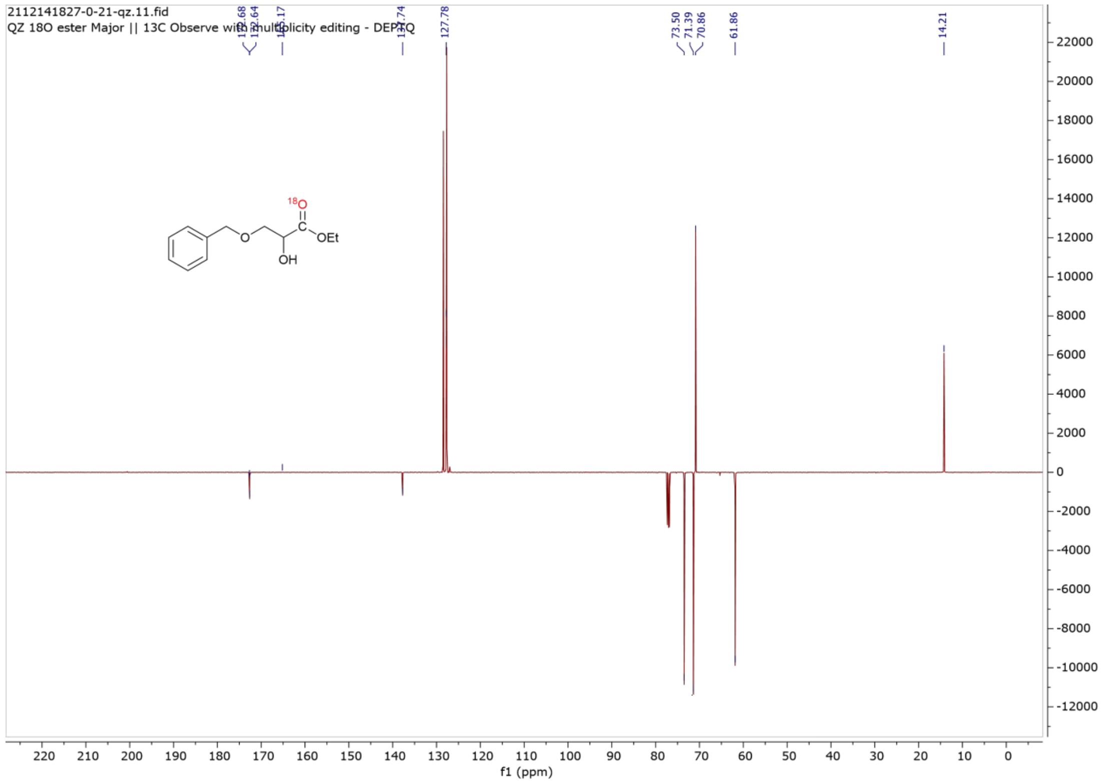

Figure S8. ^{13}C NMR of ethyl [1-^{18}O]-3-(benzyloxy)-2-hydroxypropionate in $CDCl_3$.

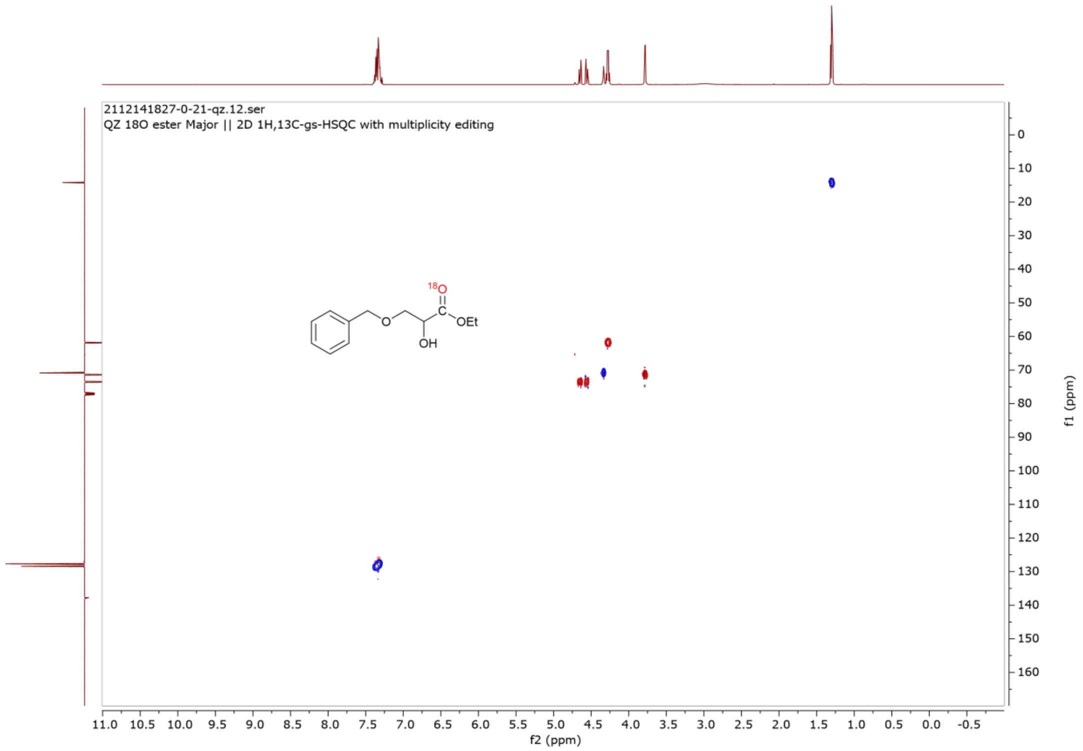

Figure S9. ^{1}H,^{13}C-HSQC of ethyl [1-^{18}O]-3-(benzyloxy)-2-hydroxypropionate in $CDCl_3$.

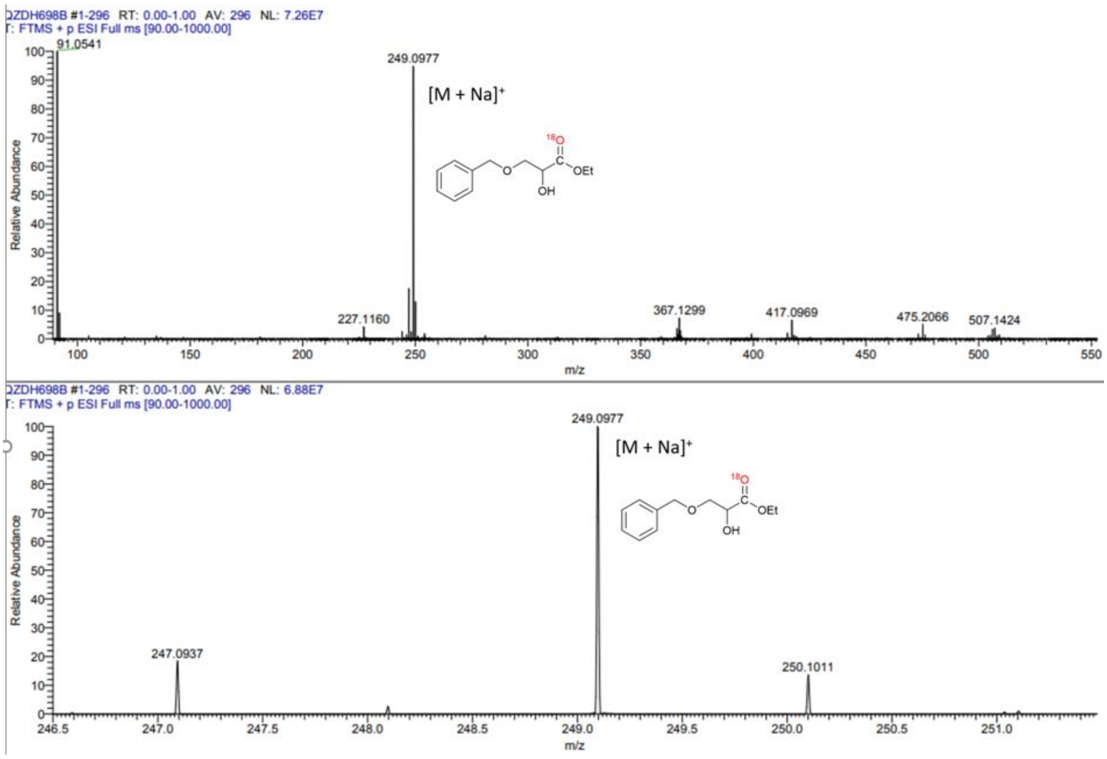

Figure S10. HRMS of ethyl [1-^{18}O]-3-(benzyloxy)-2-hydroxypropionate. The lower trace is an expansion of the upper trace and indicates ~82% oxygen -18 incorporation.

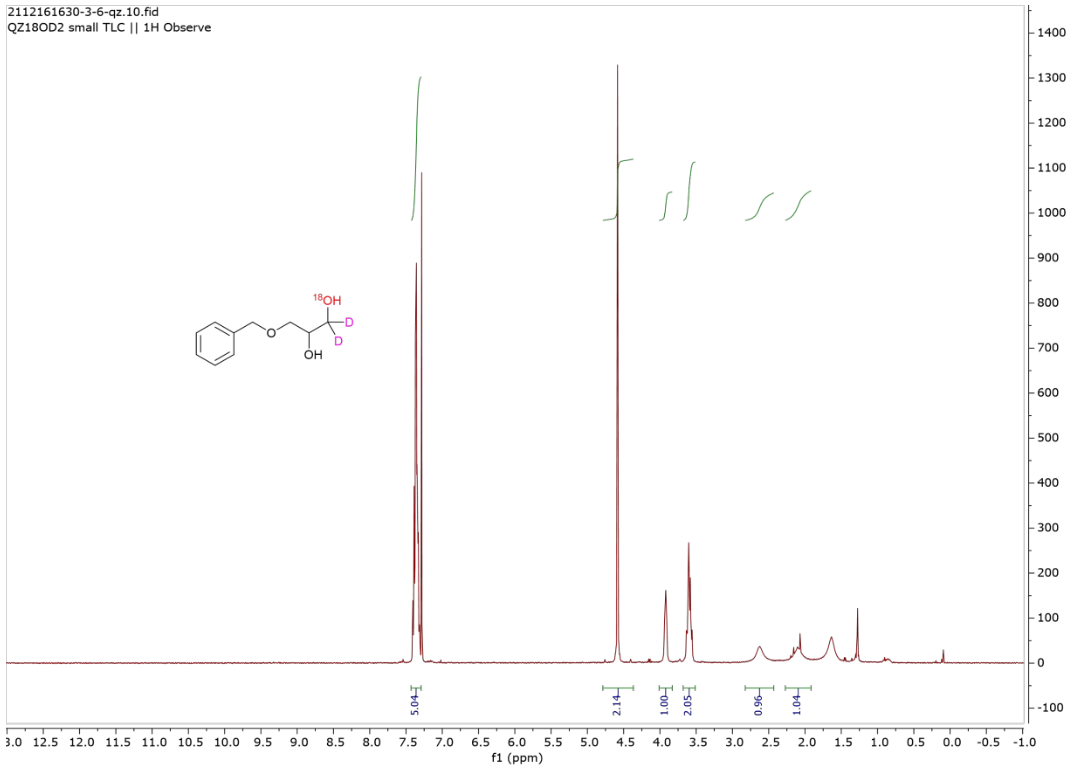

Figure S11. ^{1}H NMR of [1-^{18}O,$^{2}H_2$]-3-Benzyloxy-2-hydroxypropanol in $CDCl_3$.

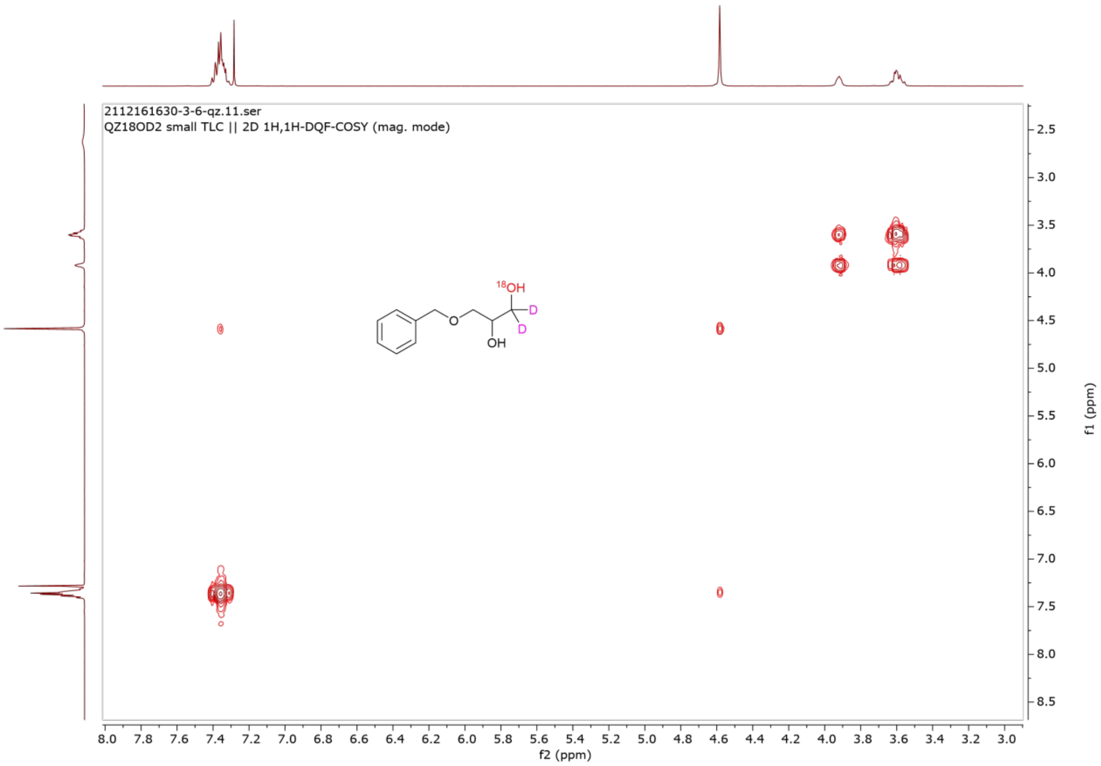

Figure S12. ^{1}H-^{1}H COSY of [1-^{18}O,$^{2}H_2$]-3-benzyloxy-2-hydroxypropanol in $CDCl_3$.

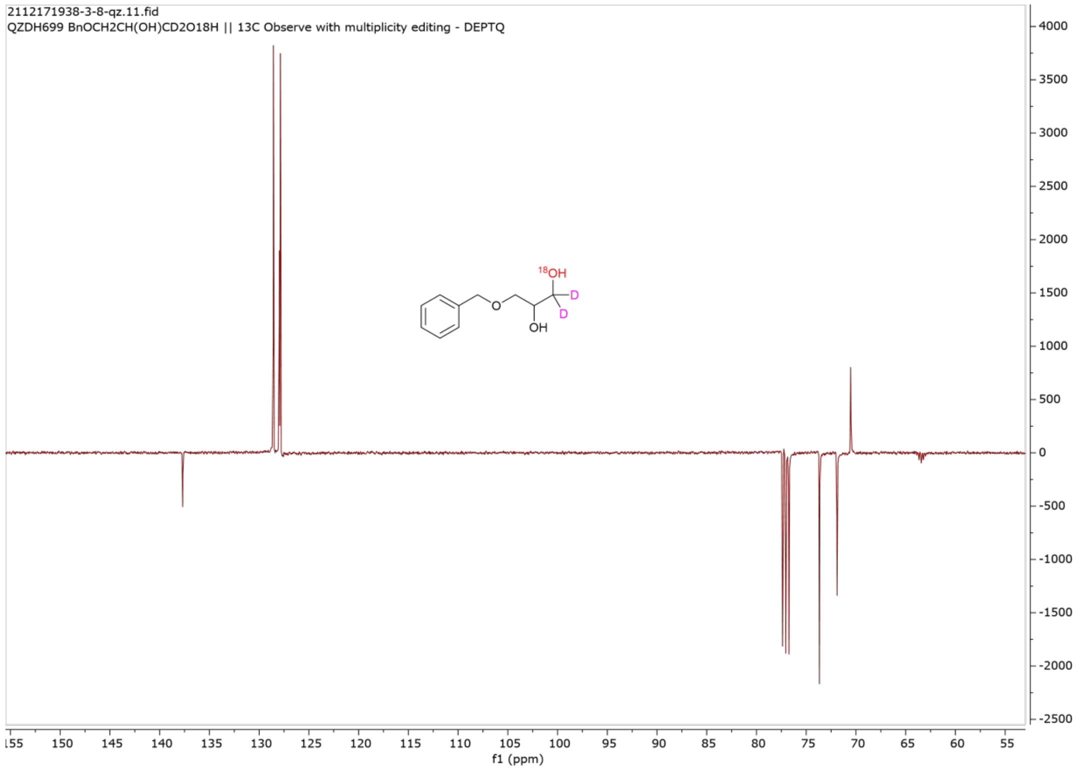

Figure S13. ^{13}C NMR of [1-^{18}O,$^{2}H_2$]-3-benzyloxy-2-hydroxypropanol in $CDCl_3$.

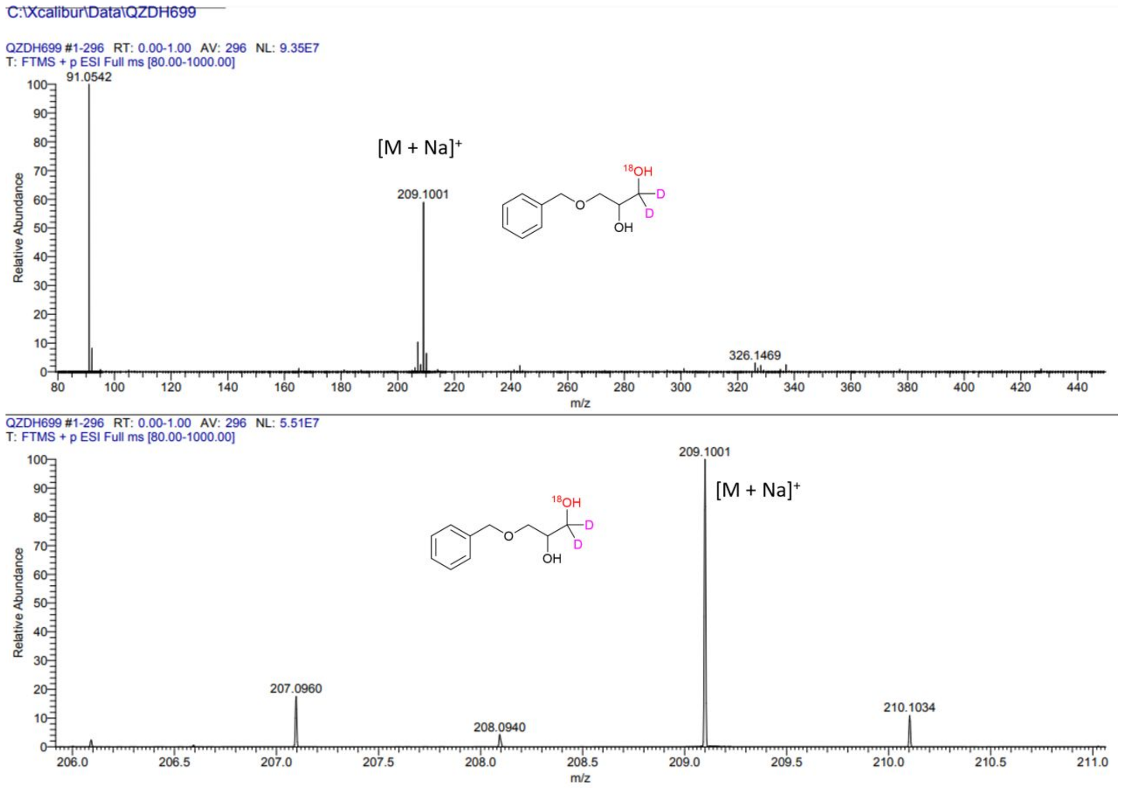

Figure S14. HRMS of [1-^{18}O,$^{2}H_2$]-3-Benzyloxy-2-hydroxypropanol. The lower trace is an expansion of the upper trace and indicates ~82% oxygen-18 incorporation and ~ 97% $^{2}H_2$.

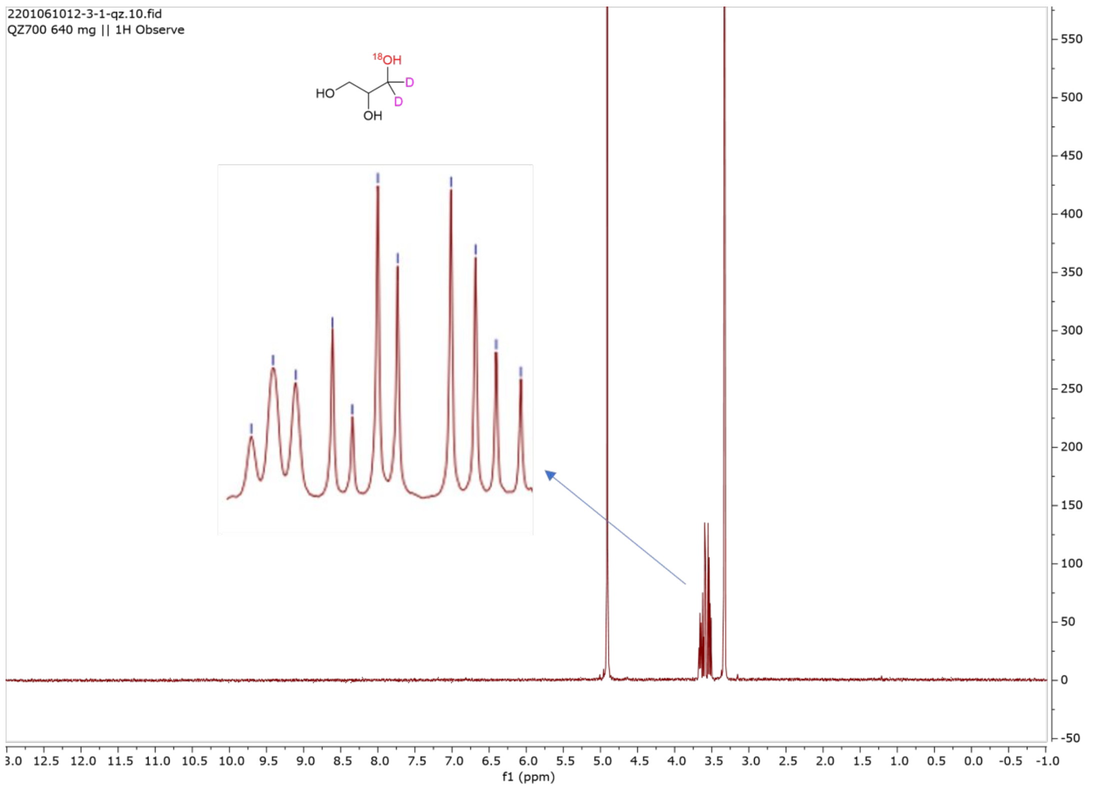

Figure S15. ^{1}H NMR of (+/-) [1-^{18}O, 1-^{2}H$_2$]-glycerol **14** in CD_3OD.

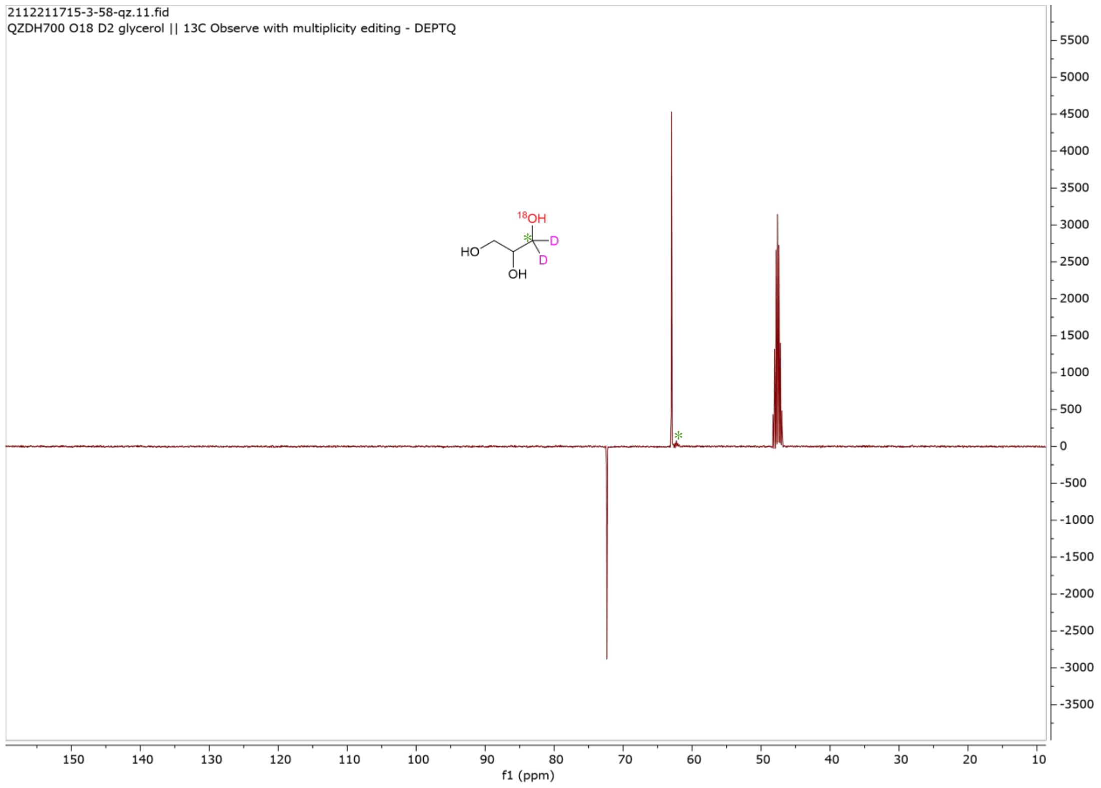

Figure S16. ^{13}C NMR of (+/-) [1-^{18}O, 1-^{2}H$_2$]-glycerol **14** in CD_3OD.

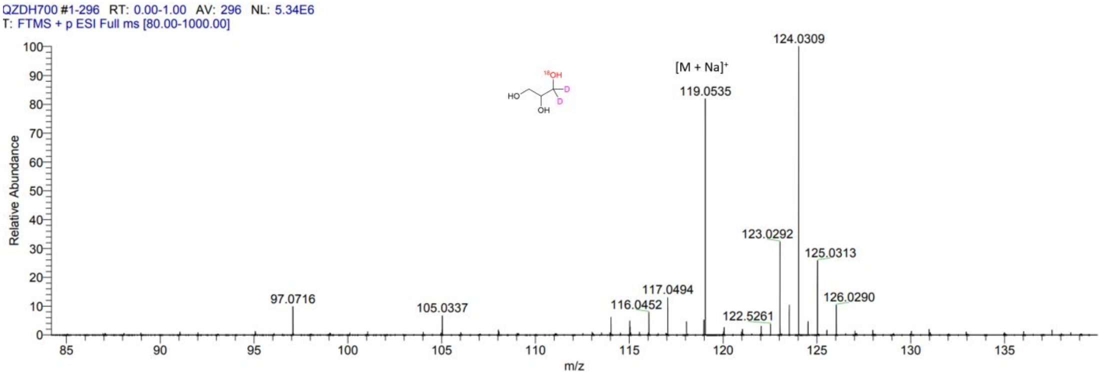

Figure S17. HRMS of (+/-) [1-^{18}O, 1-$^{2}H_2$]-glycerol **14** ~82% oxygen -18 incorporation and ~97% $^{2}H_2$.

HRMS analysis of (+/-) [1-^{18}O, 1-$^{2}H_2$]-glycerol 14 supplementation culture extracts

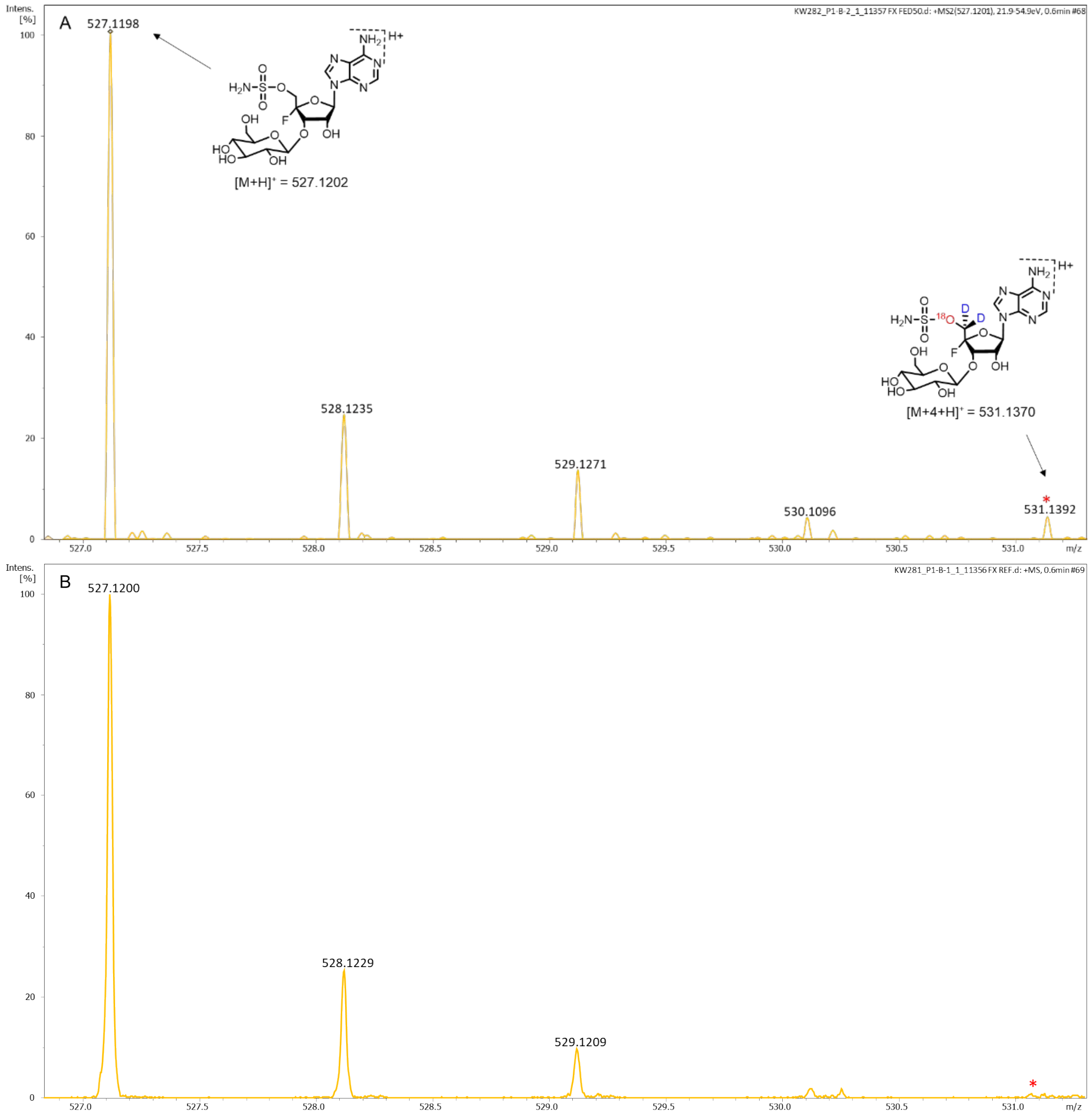

Figure S18. HRMS analysis of F-Met II **6** from (+/-) [1-^{18}O, 1-$^{2}H_2$]-glycerol **14** supplied (A) and normal glycerol supplied (B) culture extracts.

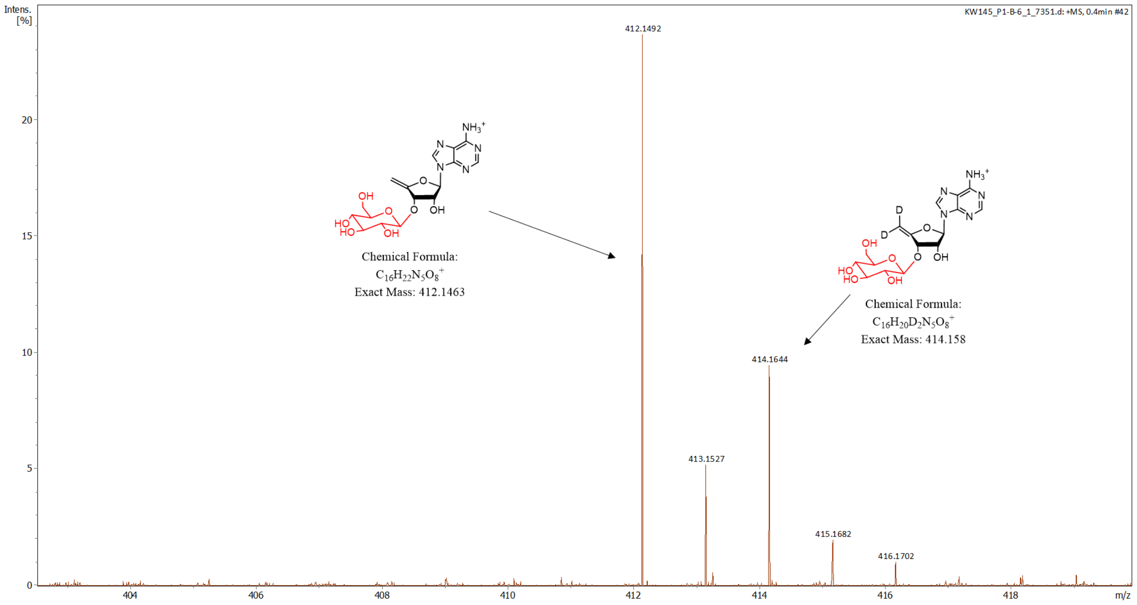

Figure S19. HRMS analysis of **13** from (+/-) [1-^{18}O, 1-^{2}H$_2$]-glycerol **14** supplied. [M+2+H]$^+$ isotopic ion of **13** was higher (8% more) than the normal glycerol supplementary culture.

Reference

S1. Perrone, P., Daverio, F., Valente, R., Rajyaguru, S., Martin, J. A., Lévêque, V., Le Pogam, S., Najera, I., Klumpp, K., Smith D. B., McGuigan, C., *J. Med. Chem.*, **2007**, *50*, 5463 - 5470.

S2. Siskos, A. P., Hill, A. M., *Tetrahedron Lett.*, **2003**, *44*, 789 - 792
